# Prevalence and Characteristics of Eagle’s Syndrome in a Syrian Population: A Cross-Sectional Descriptive Study

**DOI:** 10.7759/cureus.44853

**Published:** 2023-09-07

**Authors:** Nuraldeen M Al-Khanati, Dunia H Taha, Zafin Kara Beit

**Affiliations:** 1 Department of Oral and Maxillofacial Surgery, Faculty of Dental Medicine, Damascus University, Damascus, SYR; 2 Department of Oral and Maxillofacial Surgery, Faculty of Dentistry, Syrian Private University, Damascus, SYR; 3 Department of Pediatric Dentistry, Faculty of Dental Medicine, Damascus University, Damascus, SYR

**Keywords:** temporomandibular disorders, head and neck pain, differential diagnosis, orthopantomogram, stylohyoid syndrome

## Abstract

Objectives

Elongation of the styloid process causes different symptoms with varying degrees of severity in some people. This elongation can be detected haply during routine reading of panoramic X-ray. The aim of this study is to determine the prevalence rate of Eagle’s syndrome among a population from Damascus, Syria, and to evaluate the accompanying clinical symptoms.

Methods

This is a descriptive epidemiological study including 3,962 patients who attended one of the many dental clinics of educational and/or healthcare institutions in Damascus. All digital panoramic radiographs were reviewed, and the styloid processes were measured and assessed for elongation. Patients with styloid process of more than 3 cm in length were contacted and their consents were obtained to participate in the study to assess the presence of clinical symptoms.

Results

Radiographic elongation of the styloid process was found in 179 out of the 3,962 assessed dental records (prevalence rate was 4.5%). Length ranged from 30 to 90 mm, with an average of 55.9 mm. Of the patients with elongated styloid process, 10.2% had undergone tonsillectomy. All patients who underwent tonsil surgeries were symptomatic with at least three of the studied symptoms. Symptoms included peri-orbital pain (40.9%), orbital pain (35.2%), neck and shoulders pain (52.3%), ipsilateral headache (58%), earache (30.7%), tinnitus (37.5%), articular clicking (29.5%), throat pain (31.8%), globus sensation (22.7%), and dysphagia (21.6%).

Conclusions

The prevalence of Eagle’s syndrome according to this study was 4.5%. Patients who had undergone tonsillectomy were more likely to be more symptomatic. The most common symptom was severe headache (migraine) on the same side of styloid elongation. Panoramic radiography is a low-cost means that can be helpful in the diagnosis of vague pain and symptoms caused by Eagle’s syndrome in the head and neck regions.

## Introduction

The styloid process is a small bony projection emerging from the temporal bone, immediately anterior and slightly medial to the stylomastoid foramen. It crosses the anatomical structures adjacent to the pharynx, e.g., internal carotid artery, internal jugular vein, maxillary artery, glossopharyngeal nerve, vagus nerve, and branches of both the trigeminal and facial nerves, which travel medial to the styloid process [1].

Eagle's syndrome can be defined as the symptomatic elongation of the styloid process or mineralization of the stylohyoid ligament complex, as described by Watt Weems Eagle in 1937 [2]. Two different forms of the syndrome were known: first, classic styloid syndrome, presenting uncharacteristic pain and foreign-body sensation post-tonsillectomy, and, second, the stylo-carotid artery syndrome, which, in some cases, can be responsible for neurologic symptoms including transient ischemic attack and stroke [3].

This syndrome may cause pain in the head and neck region associated with the presence of an elongated styloid process with length of more than 2.5 cm [4]. Two major theories should be verified when assessing the original cause of elongated styloid process. Eagle suggested that the elongation was most often secondary to post-traumatic scarring and hyperplasia related to previous tonsillectomy [4]. Today, there have been predictions that anomalies of development and bone homeostasis may contribute to the elongation of the styloid process, including the presence of multiple ossification centers in the styloid process, embryonic mesenchymal conversion to osteoid matrix, osteoarthritic changes, and diseases of calcium-phosphate maintenance [1]. It was also suggested that ossification of the stylohyoid ligament may be hereditary in an autosomal dominant manner in some families [5].

Patients with styloid process elongation can be non-symptomatic, and the elongated styloid process can be seen accidentally during routine radiography. In symptomatic patients, symptoms range from continual pain, sensation of foreign body in the pharynx, swallowing difficulty, and ear pain [6]. When the styloid process is elongated, nerves and blood vessels may be affected, resulting in pain in the affected area [1]. Elongation of the styloid process occurs in around 4% of the human population, but causes symptoms only in a small group of them [7]. Higher percentage may be presented in populations with African genes and in women between the fourth and sixth decades of life [1]. The abnormalities of bone development may be considered as a cause of the elongation of the styloid process and calciﬁcation of the stylohyoid ligament.

Panoramic and computed tomography (CT) scans can play an important role in the diagnosis of this syndrome, as well as other radiographic techniques such as lateral jaw projection X-ray of the skull. In 1986, Langlais proposed a classiﬁcation for this syndrome based on the radiographic appearance; it was classified into three patterns [8]. Type I pattern represents an uninterrupted, elongated styloid process. Type II is characterized by the styloid process apparently being joined to the stylohyoid ligament by a single pseudoarticulation. This gives the appearance of an articulated elongated styloid process. Type III consists of interrupted segments of the mineralized ligament.

Management is usually conservative and non-surgical for patients with Eagle’s syndrome. Its goal is to reduce any muscle spasm or spastic tissue around the elongated process or calcified ligament. Other treatments may include steroid injections in the damaged tissues with varying results [9]. However, the treatment must be completed surgically in patients who do not respond to conventional treatment with surgical excision of the styloid processes or calcified ligaments. A surgical approach is taken intra- or extra-orally, often depending on the surgeon's experience and preferences related to the operator and the operation itself. The adverse effects and complications of surgery may include damage to adjacent anatomical structures, e.g., carotid artery or vagus nerve [3].

It is important for clinicians to be familiar with all the details and the exact anatomy of the head and neck, and they should have the ability to interpret all radiographic findings in the panoramic images of dental clinic patients. This is significant in the detection and diagnosis of diseases and syndromes that may be related to other specialties. Elongation of the styloid process may lead to ambiguous pain in the head, cheek, chin, and neck along with the carotid artery. Routine dental radiography can play a role in the coincidental diagnosis of Eagle’s syndrome by the dentist and radiologist. Therefore, we should note any radiologic changes from normal in the length of the styloid process, investigate the clinical symptoms that have no known cause, and try to link them with the radiographic findings to help the patient find the proper treatment.

Hereby, this study aimed to determine the prevalence of eagle syndrome among a population from Damascus, Syria, and to describe the accompanying clinical symptoms. To the best of our knowledge, the present study was the first study in the Syrian Arab Republic, which highlights this important syndrome and the possibility of diagnosis by simple diagnostic means, i.e., panoramic imaging.

## Materials and methods

This is a descriptive, cross-sectional study. Institutional review board approval was obtained, and the Research Ethics Committee of Damascus University reviewed and approved the present study protocol (registration no. 2021-1073). The methods of the current study included two stages; first, we reviewed the available radiographic archives related to patients who had attended dental clinics of the Faculty of Dental Medicine at Damascus University, dental clinics of the Faculty of Dentistry at the Syrian Private University, and four private dental clinics during the period of August 2021 to July 2022.

**Figure 1:**
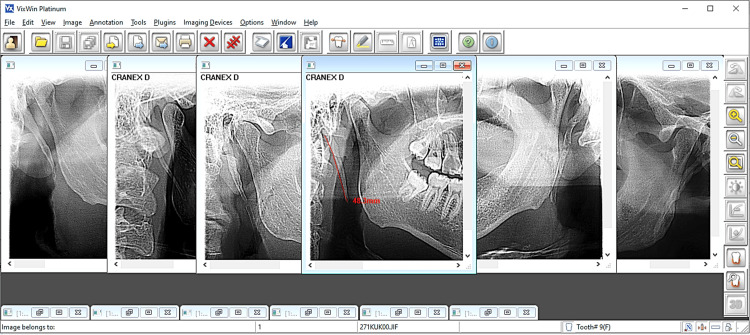
The imaging software used to calibrate, view, and interpret panoramic radiographs, and to measure the styloid process length in this study. Examples of styloid process elongation from the study sample are shown.

In order to measure the length of the styloid process, we examined the radiographic records (i.e., panoramic X-ray) of a total number of 3,962 patients. Digital dental panoramic images of male and female patients older than 16 years of age were included in this stage of the present study. All included panoramic radiographs permitted clear viewing of the styloid process from start- to end-points on both sides. A total of 3,962 digital panoramic radiographs, displayed in a 1:1 ratio, were reviewed, and VixWin Platinum imaging software v1.1 was used to calibrate images and to measure and record the radiographic length of the styloid process (Figure 1). It was measured on the apparent posterior side of the styloid process as the distance from the junction point with the tympanic plate to the tip of the process. All the radiographs were evaluated, and the measurements were taken by the same investigator. Measurements were repeated by the same researcher and by an external evaluator (i.e., oral radiologist) after an interval time of two weeks from the first measurement. Reliability analysis showed high intra-observer and inter-observer reliability. Radiographs were classified into two groups according to the presence/absence of styloid process elongation. The styloid process was considered elongated when its length was 30 mm or more. Prevalence of Eagle’s syndrome was calculated as the number of cases with elongated styloid processes divided by the total number of people in the sample.

The second phase of the current study aimed to investigate the presence of symptoms associated with the styloid process elongation. Patients with styloid process of 30 mm or more in length were contacted and their consents were obtained to participate in the clinical evaluation of associated symptoms. Informed consent was obtained from each patient who presented for clinical evaluation. The medical history was evaluated, and a clinical examination was performed for each patient. Patients were confirmed to have no history of general illness, history of maxillofacial trauma, maxillofacial infections, tumors, malocclusion, or any known causes for one of the investigated symptoms, e.g., headache, maxillofacial pain, and dysphagia. The age, gender, styloid process length, elongation side, type of elongation, history of tonsillectomy, and associated symptoms were recorded. The investigated symptoms in this study included headache, migraine, dysphagia, foreign-body sensation in throat, sore throat, otalgia, periauricular pain, supraorbital pain, infraorbital pain, eyes pain, neck and shoulder pain, articular sounds, tinnitus, dizziness, hyperacusis, and hypoacusis. Data related to symptoms were obtained from both medical records and structured interview questions.

Descriptive data were presented. Prevalence and associated symptom rates were calculated. All data were analyzed using Statistical Package for the Social Sciences (SPSS) for Windows Version 19 (IBM Corp., Armonk, NY, USA).

## Results

This epidemiological study included 3,962 patients whose radiographs were evaluated for the presence of elongated styloid processes. The elongation of the styloid process was observed in 179 patients. Thus, the prevalence rate of the styloid process elongation according to the current study was 4.5% (Table 1). Most of the elongated styloid processes showed continuous uninterrupted pattern (n=161; 89.94%). Segmented and pseudoarticulation patterns were seen in 2.79% and 7.26% of cases, respectively.

**Table 1:** Prevalence of Eagle’s syndrome (styloid process length of more than 30 mm on OPG) X^2^ = 3278.3; degree of freedom = 1; P < 0.001 OPG, orthopantomography (panoramic radiography)

Styloid process elongation (≥30 mm)	Frequency	Percentage
Absent	3783	95.48%
Present	179	4.52%
Total	3962	100%

The response rate for the clinical part of the present study was 49.2%, where we were able to contact 88 patients (out of 179), obtain their consents to participate in the study, and evaluate the symptoms associated with the styloid process elongation.

**Figure 2:**
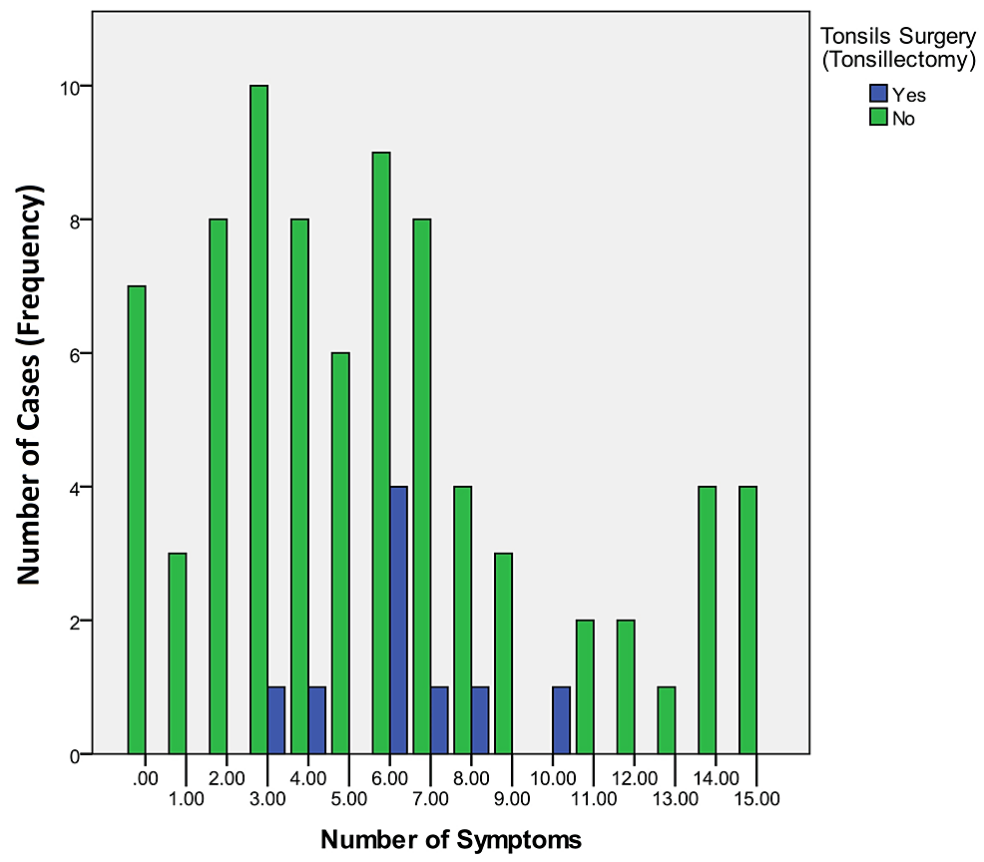
Distribution of the study sample based on the number of associated symptoms and history of tonsillectomy

The sample included 88 patients (age range: 17-74 years) with an average age of 41 years (±13 years). More than half of the sample (58 patients; 65.9%) were ≥35 years of age. Males in the studied sample were slightly more than females (Table 2). The lengths of styloid process that were measured on panoramic radiographs of the sample of the current study ranged from 30 to 90 mm, with an average of 55.9 mm (±12.7). Most of the cases with elongated styloid process (≥30 mm) showed symptoms with a wide variety in number and severity between cases. Only seven (8%) patients with styloid process elongation were completely asymptomatic. The percentage of patients who underwent tonsillectomy in the sample was 10.2% (Table 3). All patients who had previously undergone tonsillectomy had at least three symptoms out of all the symptoms investigated in the current study (Figure 2), and 18 patients (20.5%) had less than three symptoms of the studied symptoms (Figure 2).

**Table 2:** Distribution of the study sample according to gender X^2^ = 0.727; degree of freedom = 1; P = 0.394

Gender	Frequency	Percentage
Male	48	54.55%
Female	40	45.45%
Total	88	100%

**Table 3:** Distribution of the study sample according to the history of tonsillectomy X^2^ = 55.68; degree of freedom = 1; P < 0.001

History of tonsillectomy	Frequency	Percentage
Yes	9	10.23%
No	79	89.77%
Total	88	100%

**Figure 3:**
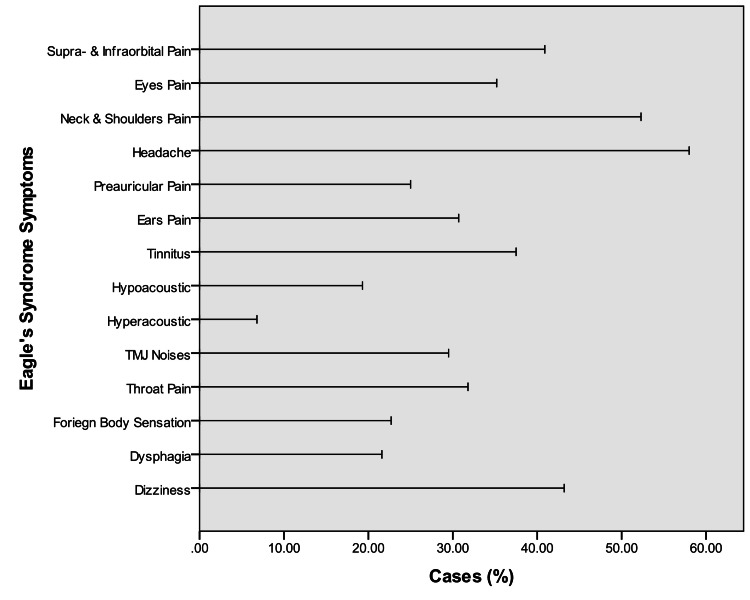
Symptoms associated with styloid process elongation

Figure 3 shows that 36 (40.9%) patients had peri-orbital pain, i.e., supra-orbital and sub-orbital pain, 31 (35.2%) patients had pain in their eyes, 46 (52.3%) patients had neck and shoulder pain, and 51 (58%) patients had severe headache on the ipsilateral side.

Some patients in the study sample complained of auricular and joint symptoms at the ipsilateral side of styloid elongation. These included pre-ear pain in 25% (22 patients), ear pain in 30.7% (27 patients), tinnitus in 37.5% (33 patients), hypoacusis in 19.3% (17 patients), and hyperacusis in 6.8% (six patients). Also, 26 (29.5%) patients reported having articular noises or clicking when opening and/or closing their mouths (Figure 3). Reported symptoms also included the following: sore throat in 31.8% (28 patients), foreign body sensation in the throat in 22.7% (20 patients), and dysphagia in 21.6% (19 patients). In addition, 38 (43.2%) patients complained of unexplained vertigo or dizziness (Figure 3).

## Discussion

Chronic pain in the head and neck area is common, and the causes of pain can vary significantly. However, when a patient experiences long-term pain in the head and neck region, dentists and physical therapists should consider Eagle's syndrome among potential causes. The initial description of Eagle's syndrome was provided by Watt Weems Eagle, who attributed the presence of this syndrome to elongation of the styloid process exceeding 2.5 cm [4]. According to Lindeman, the normal length of the styloid process ranges from 2 to 3 cm [10]. Little elongation of the styloid process (styloid process length of 30 mm) can cause symptoms. This might be due to anatomical variations and malarrangements of structures in relation to the styloid process in the area [4]. Therefore, a styloid process of 3 cm or more in length was adopted to include patients in the current study sample. Eagle found that 4% of individuals had elongated styloid processes [4], which aligns with the current study's findings, where the prevalence of Eagle's syndrome was found to be 4.5%.

Other studies reported widely varied rates [11-28]. Prevalence of styloid process elongation in different countries ranged from 0.4% to 81% (Table 4). More and Asrani conducted a study on 500 digital panoramic images and found that the average length of the styloid process was approximately 25.5 mm, and elongation of the styloid process was observed in 19.4% of the studied images [11]. Roopashri et al., who studied 300 patients aged between 10 and 70 years, found that 107 of them had elongation of the styloid process (prevalence rate of 35.6%) [12]. According to another study involving 1,135 patients aged 18 to 91 years, the rate of new cases was 30% [13]. On the other hand, the incidence rate of Eagle’s syndrome was reported to be as low as four to eight per 10,000 individuals [14]. Gossman and Tarsitano, who reviewed 4,200 panoramic images, reported a prevalence rate of 1.4% [15]. They attributed this decrease to the fact that their study included only males, while females over the age of 30 are more likely to be affected [15]. Males in the current study were slightly more than females (54.5% for males). More and Asrani noted that the length of the styloid process in males is greater than in females [11]. Keur et al. found that the prevalence of elongated styloid processes is equal in females and males despite women experiencing more symptoms at times [13].

**Table 4:** Prevalence of styloid process elongation as reported in the literature. Table is sorted by prevalence in ascending order. SD, standard deviation; L, left side; R, right side; OPG, orthopantomography (panoramic radiography); M, males; F, females

Study, Location	Mean Age (±SD)	Method for Measurements	Total Sample Size	Cases with Styloid Process Elongation	Styloid Process Length	Prevalence
Rath and Anand 1991, New Delhi (India) [16]	-	Human skulls	232	1	L: 65 mm, R: 70 mm	0.4%
Balcioglu et al. 2009, Istanbul (Turkey) [17]	-	Cadaver; OPG	22; 250	2; 10	>30 mm	3.3%
Ilgüy et al. 2005, Istanbul (Turkey) [18]	43.0 (±14.0)	OPG	860	32	30-105 mm	3.7%
Bozkir et al. 1999, Adana (Turkey) [19]	57.6 (±3.42)	OPG	200	8	39-67 mm	4.0%
Al-Khanati et al. 2023, Damascus (Syria) (current study)	41.2 (±13.4)	OPG	3962	179	30-90 mm	4.5%
Vadgaonkar et al. 2015, Karnataka (India) [20]	-	Human dry skulls	110	5	37-50 mm	4.5%
Custodio et al. 2016, Belo Horizonte (Brazil) [21]	-	Human dry skulls	15	1	64-70 mm	6.6%
Aoun et al. 2020, Beirut (Lebanon) [22]	47.6 (±16.1)	OPG	489	76	>30 mm	15.5%
More and Asrani 2010, Gujarat (India) [11]	>18	OPG	500	97	>30 mm	19.4%
Swapna et al. 2021, Riyadh (Saudi Arabia) [23]	33.7 (±12.9)	OPG	300	82	>30 mm	27.3%
Alkhabuli et al. 2020, Ras Al-Khaimah (UAE) [24]	38.1 (±13.2)	OPG	3234	903	>30 mm	27.9%
Sudhakara Reddy et al. 2013, Bhimavaram (India) [25]	37.9 (±14.6)	OPG	520	154	>30 mm	29.6%
Roopashri et al. 2012, Karnataka (India) [12]	10-70	OPG	300	107	>30 mm	35.7%
Chen et al. 2022, Taichung (Taiwan) [26]	48.2 (±17.7)	OPG	486	202	30-52 mm	41.5%
AlZarea 2017, Sakaka (Saudi Arabia) [27]	≥60	OPG	198	87	31-50 mm	43.9%
Sridevi et al. 2019, Andhra Pradesh (India) [28]	M: 41.4 (±10.2), F: 38.0 (±9.0)	OPG	500	404	30-79 mm	80.8%

It was noted that the majority of patients within the current study sample were older than 35 years, with an average of 41 years. This can be explained by the fact that the styloid process elongates with age [11,12]. The current study relied on digital panoramic images; it was emphasized that panoramic images can provide an accurate depiction of styloid process elongation [12]. Thus, it was reliable in confirming the diagnosis of Eagle's syndrome.

Symptoms of Eagle's syndrome are believed to be attributed to the abnormal stylohyoid ligament's relationship with adjacent anatomical structures, including the muscular, neural, and vascular structures, including the internal jugular vein, the glossopharyngeal nerve, the vagus nerve, and the sublingual nerve. The internal carotid artery and the superior constrictor muscle (near the apex of the styloid) are also implicated. Posterior to the apex of the styloid, the visible external carotid artery branches into the superficial temporal artery and the maxillary artery [29]. The various pains experienced by patients with Eagle’s syndrome can be explained through several pathophysiological mechanisms [29,30]: (1) fracture of the styloid process leading to granulation tissue growth, which may cause compression on surrounding structures; (2) compression of adjacent nerves, including the glossopharyngeal nerve, the inferior branch of the trigeminal nerve, and the corda tympani nerve; (3) fibrous and inflammatory changes in the tendinous portion of the styloid base; (4) irritation of the pharyngeal mucosa through direct pressure or scarring after tonsillectomy (involving nerves V, VII, IX, and X); and (5) compression or impingement on the carotid vessels, consequently irritating the sympathetic nerves within the arterial sheath.

The results of the current study indicated that the majority of patients (89.8%) could be classified under the second pattern of Eagle syndrome, which occurs with no history of tonsillectomy, as described by Eagle. The accompanying symptoms for this pattern can be explained by the impingement of the elongated styloid process against the internal carotid artery on the affected side, leading to intermittent headaches in the temporal, parietal, or frontal regions, ear pain, and episodes of dizziness. Palpation of the carotid artery might induce discomfort. The elongated styloid process can potentially compress the internal carotid artery, causing headaches in the parietal area and the region above the mastoid process [31]. When the visible external carotid artery is irritated, the patient may experience radiating pain in the area beneath the mastoid process on the corresponding side [32]. Differential diagnoses can include mastoiditis, myofascial pain, migraine, glossopharyngeal and trigeminal neuralgia, cervical facet joint pain, salivary gland dysfunction, and temporal or cervical arthritis [14,30].

It was found that elongation of the styloid process, calcification of the stylohyoid ligament, or elongation of the hyoid bone leads to a decrease in the distensibility of the stylohyoid complex, thereby impeding the natural up-and-down movement of the hyoid bone during neck and mouth motions [33]. This may result in cervicofacial pain due to the compression and irritation of the vascular and neural bundles surrounding the stylohyoid complex. Additionally, the pain in Eagle's syndrome can be attributed to provoked inflammation following stimulation of nearby nerves by the elongated styloid process, suggesting the pharmacological non-surgical treatment options as a primary treatment approach [9].

Study limitations

The significance of the current study lies in being the first study in the Syrian Arab Republic to shed light on this important syndrome, which can be diagnosed by routine radiographic imaging, allowing dentists to identify and refer it to the specialist. However, this study had some limitations. Routine panoramic radiography can occasionally detect undiagnosed diseases and silent lesions [34]. Although it provides an affordable useful diagnostic tool, the inherent image distortion and magnification are among the main limitations of dental panoramic image [35]. Thus, one should take this into consideration, and image calibration is necessary before length measurement. In this way, dental panoramic X-ray image can provide a cost-effective alternative for detecting elongated styloid processes to other imaging techniques (e.g., CT and CBCT), which are considered more precise in measurements, but with relatively more radiation dose [36]. Secondly, this study took place in a specific geographic scope, and its results are not necessarily generalizable to other regions. Thirdly, it is difficult to ensure that all reported symptoms are necessarily directly related to the styloid process elongation. Nevertheless, we tried our best to exclude other possible reasons during medical history and clinical evaluations. Another limitation was related to the study design. This was a cross-sectional descriptive study with no potential to establish a cause-and-effect status or detect changes over a period of time. Future studies that can establish a clear relationship between Eagle’s syndrome and other factors, such as temporomandibular disorders, are warranted on future scope.

## Conclusions

The prevalence rate of elongated styloid process in Damascus city, according to the current study, is 4.5%. Patients who have undergone tonsillectomy surgery have a clear likelihood of symptomatic Eagle's syndrome. An increase in the prevalence of elongated styloid process in Eagle's syndrome was observed with age. The most accompanying symptom of the syndrome was severe headache localized to the side of elongation. Eagle's syndrome must be considered as a differential diagnosis for temporomandibular joint disorders or could be regarded as a contributing factor in exacerbating these disorders. The current study indicated that patients within the sample exhibited numerous symptoms corresponding to temporomandibular joint disorders. Eagle's syndrome should be considered in the differential diagnosis of cases of migraine, various facial pains, and unexplained neck pains. Digital panoramic images can be utilized for diagnosing Eagle's syndrome, offering a cost-effective technique compared to more expensive and complex diagnostic methods.
